# “OMIC” tumor markers for breast cancer: A review

**DOI:** 10.12669/pjms.315.7627

**Published:** 2015

**Authors:** Naila Irum Hadi, Qamar Jamal

**Affiliations:** 1 Dr. Naila Irum Hadi, MBBS, MPhil, PhD fellow. Professor of Pathology, Ziauddin University, Karachi, Pakistan; 2 Dr. Qamar Jamal, MBBS, MPhil, PhD. Professor of Pathology, Ziauddin University, Karachi, Pakistan

**Keywords:** Biomarker, Breast cancer, Genomics, Proteomics, Metabolomics, ASCO – American Society of Clinical Oncology, ER – estrogen receptor, PgR – progesterone receptor, HER2 – human epidermal growth factor receptor 2, CA – cancer antigen, TAILORx – Trial Assigning Individualized Options for Treatment (Rx), MINDACT – Microarray InNode Negative Disease may Avoid ChemoTherapy using Mammaprint–, FDA – Federal Drug Authority, GC-TOF-MS – time of flight, LC/ESI-MS – electrospray ionization, PCho – phosphocholine, GPCho – glycerophosphocholine, Cdx-2 – caudal type homebox 2, KiSS1 – Kisspeptin 1, KAL1 – Kallman syndrome 1 sequence, NIST – National Institute of Standards and Technology, HMDB – Human metabolome database

## Abstract

Breast cancer is a global health issue, and as the tumor burden increases, we need to come up with newer, better technologies which are convenient, cheap, rapid, sensitive with a high specificity. Technological advancements in the field of cancer biomarker has led to the development of techniques such as mass spectrometric analysis and microarray analysis in which genes, proteins and hundreds and thousands of metabolites can be identified with the emergence of genomics, proteomics and metabolomics. This research is focused on finding biomarkers for diagnosis, prognosis, staging, treatment response and targets for chemotherapy, generating a panel of markers which provide better clinical information compared to a single marker in the panel. This review briefly summarizes application of genomics and proteomics followed by key concepts and applications of metabolomics in breast cancer, with the conclusion that an integration of the three “OMIC” technologies may hold the key to future biomarker discovery.

**Sources of Data Study Selection:**

The information for this review was collected by searching the Google Scholar and PubMed database for English articles published in the period from 2002 to 2015. The search terms included “biomarkers in breast cancer” along with the following search terms: “genomics”, “proteomics”, “metabolomics”, “breast cancer”, “mass spectrometry”, “molecular markers” and “cancer biomarker”. We have endeavored to quote only the primary sources. Titles and abstracts of retrieved studies were assessed first followed by selection and retrieval of selected full text articles.

## INTRODUCTION

Breast cancer (BC) is a major health issue in women Worldwide as well as in Pakistan. However marked geographic variation has been noted in the incidence, natural course of the disease as well as survival statistics. This is reflected in the comparison between age standardized rates (ASR) and survival rates (SR) of North American women with that of Pakistani women i.e. ASR of 99.4 per 100,000 and SR of 80%^1^ for the former with ASR of 69.1 per 100,000^2^ and SR of less than 40% for the latter.^1^ The gravity of the situation further increases when it is noted that BC in Pakistan affects younger women with an advanced stage at the time of presentation.^3^

This puts an enormous burden on the resources of a poor country like Pakistan. Mammography for screening, histopathology and blood tests for diagnosis, prognosis and treatment are considered gold standards for breast cancer.^4^ According to 2007 recommendations of ASCO for tumor markers ER, PgR and HER2 expression in primary invasive breast cancer should be evaluated for diagnosis or recurrence especially as a guide for therapy, while increasing levels of CA 27.29 or CA 15-3 may indicate treatment failure.^5^ This however cannot be applied to all breast tumors leaving a wide gap in our understanding of this heterogeneous tumor. Hence development of new methods for exploring the molecular pathogenesis of this disease becomes imperative. Detection of malignancy by a sample blood test for identification of tumor markers has been explored thoroughly in medical field. These biomarkers are released by the tumor itself or by other tissues as a reaction to the tumor or inflammation occurring in response to tumor. An ideal tumor marker is easily measured, reliable, and cheap, with a high sensitivity and specificity. It should help not only in screening early cancer but also recurrence, vary with different stages of disease and has prognostic and predictive value.^6^

This review briefly covers the concepts of genomics and proteomics, followed by an in-depth analysis of the evolving field of metabolomics for biomarker discovery in breast cancer.

### 1. “Omics” in Breast Cancer

In the quest for identification of a suitable biomarker for breast cancer, novel and high-yield technologies like genomics, proteomics, and metabolomics have received a lot of attention in recent times, prompting the researchers to name this as the era of “Breast cancer – OMICS”.^7^ Studies have shown that malignant transformation of normal breast tissue and evolution of metastatic clone involves altered gene expression (altered transcription) or altered protein expression (altered translation).^8^ This has led to the development of promising technologies of genomics, proteomics (respectively) and metabolomics.

Gene expression profiles of certain predictive and prognostic markers of breast cancer have been developed and are available commercially such as molecular technologies for improvement in breast cancer diagnosis and treatment with the development of MammaPrint (Agendia, Amsterdam, The Netherlands) (a 70 gene microarray study for prediction of breast cancer relapse)^9^ and OncotypeDx (Genome Health, Redwood city, CA, USA) (a 21 gene expression profile by RT-PCR).^10^ These have been approved by FDA and are commercially available since 2007 and 2004 respectively. Their value in assessing individualized options for treatment in selection of an effective and appropriate chemotherapeutic agent for breast cancer patients is being explored in ongoing clinical trials: TAILORx^11^ and MINDACT,^12^ but their routine clinical use is not yet recommended.^13^

Recently researchers are interested in finding out whether other ‘omic’ technologies can also add to the information provided by genomics.^14^ Elevated levels of protein in biological fluid in cancer can be due to atypical secretion, shedding of membrane-associated proteins, change in cancer cells polarity, increased expression of proteases^15^ and single nucleotide polymorphism (SNP) of signal peptide,^16^ etc. HER2 is a cancer biomarker, and classic example of a membrane bound tyrosine kinase, that is shed into fluids. HER2 protein has an extracellular domain (ECD), a transmembrane domain and a cytoplasmic domain. The ECD of HER2 is cleaved by a protease from the receptor protein and can be detected as a biomarker in serum. Over expression of HER2 seen in some cases of breast cancer is an indicator of poor prognosis for these patients.^5^ Since 2000, HER2 test has been approved by FDA and is used in the management and follow-up of patients with metastatic breast cancer.

mRNA transcript does not reflect the function of proteins, hence different proteomic strategies in biomarker discovery have emerged as proteins in complex mixtures require systemic characterization by mass spectrometry (MS). Limitation of MS for proteomic approaches include improper sample collection and storage, inability to identify established serological biomarkers, bias in identification of high-abundance molecules within the serum, conflict in reporting of ms peaks reported by different research laboratories^17^,^18^ and possible artifacts in bioinformatics.^19^ Hence serum proteomic analysis and profiling is not currently recommended for clinical use by experts.^13^

Enzymes are proteins, and there should be good quantitative relationship between mRNA concentration and enzyme function. But on the contrary, metabolites which are downstream are better indicators of enzyme activity,^20^ and are more sensitive monitors of a change in biological system, represented by the genome (‘genomics’), transcriptome (‘transcriptomics’), proteome (‘proteomics’) and metabolome (‘metabolomics’). At the end of the spectrum, metabolome represents the phenotypic changes and even slight alterations in metabolites can be detected.^21^ Hence cancer researchers have renewed interest in the field of metabolomics for the discovery of specific biomarkers for use as diagnostic or prognostic markers.^21^

### 2. Metabolomics

Warburg effect (put forward by Otto Warburg in 1924)is characterized by an increase in glucose uptake by cancer cells converting it into lactate by glycolysis in spite of normal oxygen supply, hence also called ‘aerobic glycolysis’ or ‘aerobic fermentation’.^22^ Cancer cell is also shown to have an altered protein metabolism and altered lipid metabolism.^23^ Hence cancer is regarded as a disease with gene mutation resulting in changes in gene expression to produce a metabolic phenotype with altered glycolytic, amino acid, nucleotide and glycerophospholipid / lipid metabolism. This results in a cancer cell phenotype resulting in cancer cell growth, differentiation and survival.

Metabolomics technology involves identification of hundreds to thousands of metabolites and exploring multiple cellular pathways at the same time. Presence of small molecules in the body fluids or tissues contribute to the construction of a unique ‘fingerprint’, distinguishing between disease and health implying that metabolomics can distinguish between cancer and normal tissues. So metabolomics is emerging as a promising new ‘omics’ field^7^ a high throughput technology increasingly being used for breast cancer research especially for screening, diagnosis, cancer typing, staging and therapeutic intervention.^21^,^24^ Advantages of metabolomics include being cost-effective, high throughput, and being automated with sample analysis taking 10 to 30 minutes per sample approximately.^23^

Two terms are frequently used in metabolomics; ‘metabolic profiling’ and ‘metabolic fingerprinting’. Metabolic profiling refers to a measure of total number of individual metabolites in a sample while metabolic fingerprinting means measuring a group or class of metabolites or quantification of a limited number of metabolites to differentiate between different samples.^25^ In spite of recent advances in the field of metabolomics its application has been limited by technical problems.

### 2.1 Metabolomic Approaches

Most popular methods for metabolomics include mass spectrometry (MS) and nuclear magnetic resonance (NMR) spectroscopy,^26^ which are complementary to each other. Mass spectrometry can be coupled with gas chromatography (GC-MS) (identification of approximately 1000 metabolites), with liquid chromatography (LC-MS) (identification of hundreds of metabolites) or capillary electrophoresis (CE-MS). NMR MS identifies a variable number of metabolites depending on the nature of samples i.e. 20 – 40 metabolites in tissue samples and 100 – 200 in urine samples.^20^,^25^ GC-MS and LC-MS are commonly used techniques for cancer samples.^21^

Benefits of NMR include high reproducibility, ability to quantify metabolites in complex mixtures and metabolite detection in vivo, as well as in biological fluids and tissues without any prior preparation of the sample. Its main disadvantage is its low sensitivity.^27^ An improvement of NMR spectroscopic procedure is a technique called high resolution magic angle spinning (HR-MAS) NMR spectroscopy, which involves spinning of a biopsy sample at an angle to the magnetic field, to improve the spectrum resolution.^28^ Its advantage include simultaneous in situ assessment of both aqueous and lipid soluble metabolites.^23^ High resolution NMR (HR-NMR) and HR-MAS MRS can be used on biofluids and tissues as they do not cause destruction of samples making it possible to carry out parallel analysis.^29^

Benefits of GC-MS include high sensitivity, quantification of metabolites and ability to identify more compounds in comparison to other MS techniques. Its main limitation is complex and lengthy steps involved in sample preparation and interpretation of its spectra. Benefits of LC-MS is its use for non-volatile compounds, quantification of wide range a of metabolites and its complementary nature to GC-MS.^21^

CE-MS separates and identifies polar or ionic compounds in complex mixtures, has high resolution with no complex and laborious sample handling as for GC-MS, and has low sensitivity but high variability than that of LC-MS or GC-MS.^21^

### 2.2 Diagnosis, Prognosis & Treatment of Breast Cancer

Heterogeneity of breast cancer (BC) ranges from its morphology, to prognosis, to metastatic potential and to treatment response. Studies carried out for understanding breast cancer pathogenesis are aimed at identification of biomarkers and new targets for effective cancer chemotherapy.^30^ A GC-TOF MS based metabolomics study in breast cancer detected 368 metabolites that differentiated between cancer and normal tissues, a property that can be utilized for screening of breast cancer. The ratio of cytidine-5-monophosphate / pentadecanoic acid was the most specific and sensitive discriminator.^31^ In a research carried out to observe changes in lipid metabolism, an important feature of cancer, increased levels of sn-glycerol-3 phosphate was detected by GC-MS, and increased levels of phospholipids by LC-MS in breast cancer tissue.^32^

**Fig.1:**
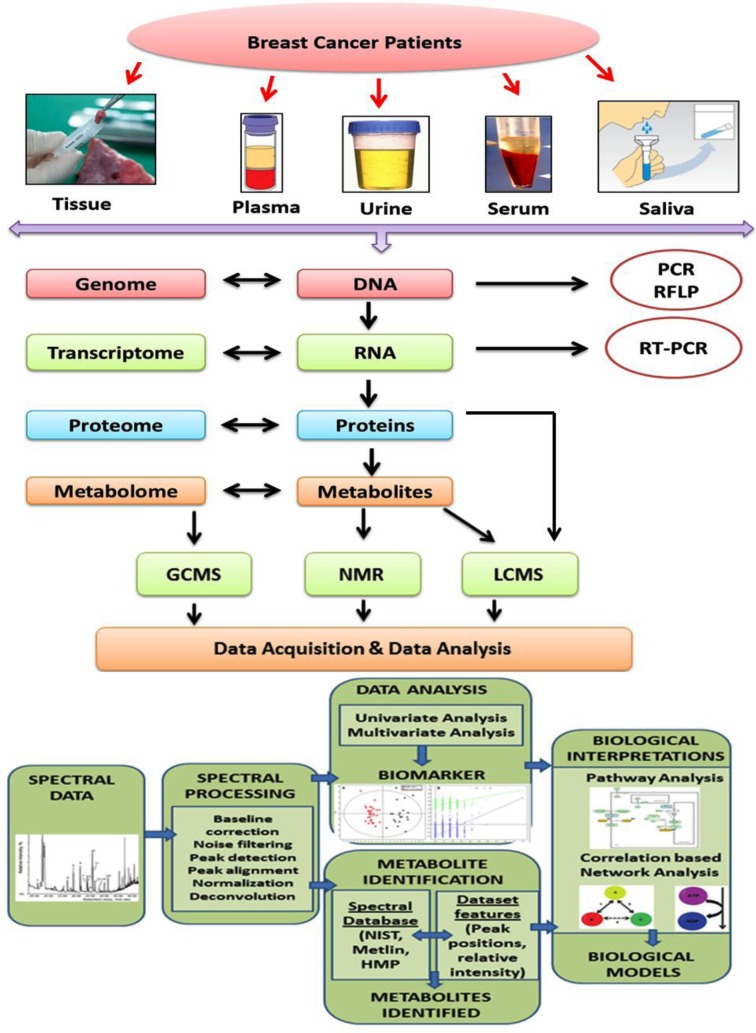
Schematic representation of “-Omic” platforms in breast cancer biomarker discovery.

Breast cancer prognosis can be predicted by analyzing the metabolic profile and comparing with survival rates. Giske degård et al.^33^ analyzed breast cancer tissue by HR-MAS MRS and found high levels of glycine and lactate in a subgroup of ER positive breast cancer patients with lower survival rates and hence poor prognosis. The other subgroup of ER positive patients had better prognosis while these metabolic changes (elevated glycine and lactate) were not seen in ER negative patients. In another study it was reported that ER positive Luminal type A breast cancer has a variable response to hormone therapy, dividing this group into responders and non-responders. Metabolomic analysis of luminal type A identified three groups with the help of ‘a’ and ‘b’ glucose amino acids, myoinositol and lipid residues. Gene analysis of one of the group of luminal A subtype showed its relationship to cell cycle and DNA repair and most probably represented the non-responders to hormone therapy.^34^

Increased levels of total choline (t-Cho) containing compounds have been detected in breast cancer cells forming a basis for many metabolomics analysis carried out in these patients.^35^-^37^ For example, in a study carried out by Mimmi et al.(2011),^36^ breast tissue biopsies from normal subjects, patients with fibrocystic disease, benignlesions and breast cancer were analyzed by LC / ESI-MS for the presence of Cho, PCho and GPCho. Results showed raised levels of choline and its phosphorylated metabolites in subjects with benign and malignant tumors only. Metastasis in breast cancer has also been detected with the help of a distinct metabolic profile in the serum or urine of these patients.^35^,^38^ Similarly serum metabolic profile by NMR showed that metastatic breast cancer can be differentiated from early stage breast cancer with a prediction accuracy of 72%.^35^ Jobard et al.^39^ carried out serum metabolomics by HNMR to differentiate between localized early disease (EBC) and metastatic breast cancer (MBC) with respect to diagnosis, prognosis and management of these patients. He constructed a model with 9 differentiating metabolites which included histidine, acetoacetate, pyruvate, glycerol, glycoprotein (N-acetyl), glutamate, mannose and phenylalanine.

Asiago et al.^38^ studied recurrence of breast cancer by a combination of analytical (NMR, GC- MS) and multivariate statistical methods to identify 11 metabolites which can predict recurrence with a sensitivity of 86% and specificity of 84%. By combining NMR and LC- MS, four metabolites namely threonine, glutamine, isoleucine and linolenic acid were found to be predictors of response to neoadjuvant chemotherapy. Breast cancer patients were placed into three groups, i.e. with no response, partial response, or complete response. Altered metabolic profiles were seen for these four amino acid metabolites which distinguished between the different groups.^40^

Pharmacometabolomics, a promising and novel field can predict response to chemotherapeutic agents. In patients with metastatic breast cancer treated with paclitaxel and lapatinib, serum metabolic profile was analyzed before and during chemotherapy, and was found to have a positive correlation with patient survival and time to progression in HER2 positive patients. Metabolomics also helped in selecting the subset of HER2 positive breast cancer patients with metastatic disease who were responsive to this combination therapy.^41^

### “Omics” in Pakistan: Present and Future

Different types and sites of BRCA 1 and 2 gene mutations, known risk factors for breast cancer, have been studied extensively in Pakistani women with BC (both sporadic as well as familial cases).^42^-^44^ Breast cancer research in recent times is focusing more on gene polymorphisms other than BRCA 1 and 2 as a possible explanation for racial differences in incidence, clinical presentations and prognosis of breast cancer. Prevalence of TP53 mutations in BRCA1 & 2 negative young Pakistani BC patients (≤ 30 years) was assessed, uncovering novel mutations which can account for a subset of cancers occurring in the younger age group.^45^ An association between vitamin D receptor Cdx-2 gene polymorphism and risk of breast cancer in premenopausal women revealed an increased risk of BC in young women with GG genotype,^46^ while another study reported a reduced expression of metastasis suppression genes (KiSS1 and KAL1) in Pakistani BC patients.^47^ Absence of FANCM c. 5101c &gt: T mutation in triple negative and BRCA 1 & 2 negative patients^48^ and insignificant role of RAD51C, a gene responsible for DNA repair and stability of genome, in BRCA 1 & 2 negative BC patients^49^ have also been reported.

Literature search regarding proteomics research in Pakistan yielded a single article reporting a distinct proteomic profile distinguishing between breast cancer, benign breast lesions and healthy controls, serving as diagnostic biomarkers. Sera of the three groups were analyzed by one-dimensional SDS polyacrylamide gel electrophoresis (PAGE) and protein identified through LC/MS/MS.^50^ Similarly in spite of global advances in field of biomarker discovery for BC by metabolomics, to the best of our knowledge no work has been carried out in Pakistan. Currently we are working on BC metabolomics biomarker project, which is in final stage of completion, in collaboration with H.E.J. research institute of chemistry, Dr. Panjwani Center for Molecular Medicine and Drug Research (PCMD), University of Karachi.

## CONCLUSION

High-throughput “omic” technologies, especially metabolomics is a promising evolving field for advancing our knowledge and understanding of breast cancer pathogenesis, identification of diagnostic biomarkers, tumor typing and staging, and response to therapy. Extensive and widespread studies employing a large sample size are required for proper validation of these different biomarkers. Clinical application of “omics” approach can further be improved by integration of genomics, proteomics and metabolomics, thus exploring new frontiers in biomarker discovery for breast cancer.
